# Views on online self-help programmes from people with eating disorders and their carers in UK

**DOI:** 10.1093/eurpub/ckab046

**Published:** 2021-07-07

**Authors:** See Heng Yim, Lucy Spencer, Gemma Gordon, Karina L Allen, Peter Musiat, Ulrike Schmidt

**Affiliations:** 1 Section of Eating Disorders, Institute of Psychiatry, Psychology and Neuroscience, King’s College London, London, UK; 2 The Eating Disorders Service, Maudsley Hospital, South London and Maudsley NHS Foundation Trust, London, UK

## Abstract

**Background:**

Digitalizing the healthcare system has been declared a priority by the UK government. People with eating disorders (EDs), especially those with bulimia nervosa (BN) or binge eating disorder (BED), and ED carers may benefit from online self-help programmes, due to the shame and stigma associated with EDs and barriers in accessing treatment, skills-training or support. Qualitative studies are needed to explore stakeholders’ needs, attitudes to and views about online self-help, to optimize intervention design and delivery.

**Methods:**

Focus groups and telephone interviews were conducted with people with BN or BED, and carers of people with anorexia nervosa, between March and September 2018 in the UK.

**Results:**

People with EDs and carers perceived online self-help positively in the context of barriers to seeking and accessing treatment and support, despite some seeing it as inferior to face-to-face support. Most reported little experience with online interventions. Participants thought the disadvantages of online interventions could be overcome by reminders, progress summaries, regular engagement and engaging with peers. Receiving guidance was seen as an important functionality in the intervention by people with EDs.

**Conclusions:**

People with EDs and their carers are aware of the potential benefits of online self-help despite having little experience with this form of intervention. A stepped-care approach that utilizes technology-based interventions as a first step and makes such interventions available directly to the consumer may fit the attitudes and needs of stakeholders. The study provides a foundation for future research on design and delivery of ED online self-help.

## Introduction

Eating disorders (EDs) negatively affect the quality of life of both people who suffer from them and their informal carers,^1^ typically family members, partners or friends. Guided self-help has been recommended by the National Institute for Health and Care Excellence (NICE) in the UK as a first step in the treatment of adults with bulimic-type eating disorders, namely bulimia nervosa (BN) or binge eating disorder (BED).^1^ Such interventions can reduce actual and perceived barriers to accessing care.^2^^,^^3^ Similarly, NICE guidelines recognize the substantial burden of people with EDs on caregivers^4^ and recommend that carers be kept informed and offered their own support. Carers, whilst highly motivated to help, often lack the skills to do so effectively.^5^^,^^6^ They also frequently report that little support is available,^7^ or is difficult to access due to time constraints, as well as concerns around privacy or stigma.^8^

Among the various forms of self-help, online self-help is particularly scalable and its interactivity and accessibility may appeal to ED patients and carers alike.^9^ Evidence for the effectiveness of online self-help for BN and BED on ED psychopathology and abstinence on binge eating and compensatory behaviours exists, but uptake and completion rates are highly variable. For example, in a systematic review from Schlegl et al.,^10^ the authors reviewed 45 studies (including 22 randomized controlled trials), and provided preliminary evidence for the effectiveness of internet guided self-help especially for BN. The completion rate of the reviewed studies ranged between 18.4% and 95.5% with a mean rate of 57.6%. Likewise for carers, there is an emerging evidence-based for the efficacy of such interventions, mainly in carers of individuals with anorexia nervosa (AN).^11^^,^^12^ Qualitative studies into the opinions of users facilitate our understanding of people’s attitudes and perceptions of online self-help, providing invaluable information to develop future online interventions. There is a relatively limited number of previous studies exploring perceptions and experiences of online self-help in EDs. These studies revealed a general positive attitude towards online self-help. Common perceived advantages include convenience, accessibility and confidentiality.^13^^,^^14^

The potential of online self-help for people with EDs and their carers merits further exploration. A recent policy paper from the UK’s Secretary of State for Health highlighted the use of technology in healthcare as a strategic priority,^15^ with the ambition to make the UK a world leader in this area. Understanding stakeholders’ views on online self-help for EDs will support this policy direction.

To our knowledge, this is one of the first qualitative studies that investigates both people with EDs and their carers’ views on online interventions. In parallel integrated studies, this paper aims to explore the (i) ‘experiences’, (ii) ‘needs’, (iii) ‘values and attitudes’ the two groups hold regarding ED online interventions in the context of the UK healthcare system; (iv) possible ‘under-served’ population groups; and (v) ‘facilitators and barriers’ when optimizing reach, adoption, implementation and maintenance of such programmes.

## Methods

The present two studies are part of the UK arm of two multi-country clinical trials into online treatment of people with BN or BED (Study 1) and carers of people with AN (Study 2), under the ICARE Consortium (https://www.icare-online.eu). Protocols of these studies are available.^16^^,^^17^

Ethical approvals were obtained by the UK Health Research Authority (16/NW/0888, 16/NW/0885). Participants could choose to take part in either a focus group or semi-structured telephone interview. We offered participants the option of a group discussion as some might wish to share ideas with others. Informed written consent was obtained prior to participation. Topic guides were derived from the implementation science RE-AIM Framework (http://www.re-aim.org/) (see Supplementary Appendix S1).^18^

### Procedure

For Study 1, adult females either with a clinical diagnosis of, or self-identified as suffering from BN, BED or a related sub-threshold binge/purge ED were recruited via the South London and Maudsley ED Outpatient clinic and King’s College London through posters, clinicians’ referrals and university circular emails. Previous experience of online intervention was not taken into account when assessing eligibility. Two focus groups totalling five people (*N* = 2 and 3) and 10 telephone interviews of 35–63 min duration were conducted between March to July 2018. Six participants were accessing outpatient group therapy at this time. The small focus group size resulted from the non-attendance of three individuals who signed up in advance of the meeting, but failed to attend.

For Study 2, participants were carers of individuals with AN or atypical AN who were part of the ICare study and were recruited via the online Minddistrict platform. All carers who took part had received the We Can intervention^16^ prior to participating, and any other additional experience of an online intervention was not considered. Twelve telephone interviews of 43–68 min duration were conducted between February and September 2018. One-to-one phone interviews were selected over focus groups, due to carers’ preferences for confidentiality and flexible scheduling options.

### Analysis

The interviews were audio-recorded, transcribed verbatim and anonymized for coding. Interviews and transcriptions for Study 1 and 2 were conducted by co-authors SHY and LS, respectively.

Thematic analysis^19^^,^^20^ was used to allow an inductive exploration of participants’ perceptions and opinions rather than idiographic meaning. Coding was conducted by SHY (for Study 1) and LS (for Study 2). They read and familiarized themselves with the scripts prior to coding. Codes were then grouped into themes using the NVivo software (version 12). The research team acted as ‘critical friends’^21^ to review and refine the codes and themes around areas of uncertainty for possible alternative interpretations. Codes were read over and re-grouped, themes were revised. For Study 1, the process resulted in the generation of 3 themes and 10 sub-themes. Study 2 generated 4 themes and 11 sub-themes.

## Results

Table 1 summarizes themes and sub-themes for the two studies. Supplementary tables S1 and S2 (Supplementary Appendix S2) present illustrative quotes grouped into themes.

**Table 1:** Themes and sub-themes for Study 1 and 2

Theme	Subtheme
Study 1. People with bulimic EDs	
• Barriers affecting help-seeking behaviour	• Prior experience
	• Self-stigmatization
	• Internal struggles
• Attitudes towards self-help	• An option but not a replacement of ‘real’ treatment
	• Works differently for different people
	• Contradictory thoughts and feelings
	• Empowerment versus overwhelming feeling
	• Loneliness versus safety
• Engaging with self-help interventions	• Baby steps
	• Immediate support
	• Monitoring and feedback
	• Limited effort
Study 2: Carers of People with AN	
• Current status of existing ED carer support	• Online support
	• In-person support
• Negatives of online support	• Better for ‘other people’
	• Lack of motivation
	• Practical issues
• Positives of online support	• Content
	• Accessibility and layout
	• Impact on relationship with loved one
• Carer understanding of EDs	• Causes
	• Accessing support and treatment
	• Living with an ED

### Study 1: Patients’ views

#### Barriers affecting help-seeking

Most participants had experience with online self-help information or were aware of self-help books. However, few were aware of or had accessed online self-help programmes for ED.

While some had experienced long waits in accessing NHS treatments, others simply presumed there would be a long wait, deterring them from seeking NHS treatment. Those who received help early described themselves as ‘lucky’. Others described negative experiences with NHS staff as an additional barrier towards help-seeking.

A lack of awareness of needing help and of available treatments added to the gap between seeking and receiving help. Some participants expressed internal struggles around needing and deserving help. Many mentioned feelings of shame, guilt and embarrassment in disclosing their EDs, thinking their illness was their own fault (related to lack of will power or greed), an ‘indulgence’ or ‘addiction’. These self-stigmatizing views were exacerbated by perceiving BN and BED as ‘lesser-known’, ‘less serious’ and not ‘good enough’ when compared with AN. Many found it hard to accept their struggles. As a result, some did not seek help for their EDs despite getting help for depression and anxiety.

#### Attitudes towards self-help

Although many participants acknowledged the advantages of online self-help, most regarded self-help as a prelude to getting ‘proper’ treatment (‘one tool in the toolbox’), rather than an alternative to traditional treatments. Advantages identified included being given comprehensive information, e.g. therapeutic and dietetic advice and support at the same time. Online self-help was accessible regardless of their illness severity and geographical location. Some participants were sceptical (e.g. ‘not optimistic’, ‘not having much faith’). However, the presence of a willingness to try anything was strong, especially when the person’s motivation was at its peak at the time of seeking treatment.

Participants pointed out that online self-help might work differently for different people. Some of the under-served populations include the older generation, people with learning difficulties, and those with limited internet access. Some thought online self-help might be more helpful for adults than adolescents, as it could be difficult for adolescents to stay self-motivated for the duration of the programme.

Participants expressed mixed feelings towards the functions of online self-help. On one hand, the self-directed nature of self-help was perceived as potentially empowering. They valued input from other people (peers, the online guide) on the platform who observed their progress and kept them accountable. In contrast, being given information and working on change by oneself was seen as overwhelming by some participants. Another area where there were contrasting views concerned the need for safety and solitude on the one hand versus feeling lonely on the other. Participants valued the internet as a ‘shield’ that kept them from feeling exposed. That said, human interaction was seen as important to break the isolation and the feeling of being ‘abandoned by the system’. This could be achieved by regular interactions via moderated live chat or message boards. There was a strong desire to relate to ‘someone like me’ (i.e. same gender, age, ethnicity, sexuality) on the platform. However, this raised some concerns over people making comparisons or writing unhelpful comments. For this reason, participants emphasized the need for moderating conversations.

#### Engaging with online self-help interventions

Rather than anticipating full recovery through self-help, many participants thought that the self-help programme would allow them to make small changes through setting small goals. To avoid feeling like a failure if the goals were not achieved, they hoped the programme would frame goal-setting in a positive and encouraging way. They also expected to complete regular weekly or daily tasks to keep up momentum.

Participants saw online self-help as an immediate support to be used at times when other help is unavailable. Some suggested having an ‘immediate help’ functionality for coping with acute crises.

Monitoring progress was a key feature proposed by participants. Some participants saw the platform as a space for them to log what they ate, their cravings, binges and feelings. Additionally, participants wanted a reward feature when they achieved a milestone, or having others notice or congratulate them on their success.

Many participants emphasized the importance of usability: the programme should require as little effort as possible and minimal scientific jargon. Audio, video or podcast could be used alongside textual information to enhance interactivity. Most preferred using mobile apps over the web as these allow use when ‘out and about’. For some, a small commitment of around 5–15 min per day was ideal. To facilitate integrating the programme into daily life, many emphasized the importance of integrating the app with other media channels such as emails and texts along with the use of personalized reminders. Yet, some participants cautioned that the use of reminders might be anxiety-provoking. They recommended sending reminders sparingly and using subtle reminder messages in case someone else picked up the phone.

### Study 2: Carers’ views

#### Current status of existing ED carer support

Participants identified two types of carer support—online and face-to-face—although the majority of carers had accessed little support previously (outside of searching for information online). Several carers expressed that, although they were aware of reputable sites providing anorexia-specific information, more in-depth advice would be helpful. Carers also mentioned following social media accounts of health charities and individuals who promoted recovery, watching documentaries relating to EDs, and looking for more in-depth news or research articles.

A minority of carers reported that they had received face-to-face support (e.g. peer-led carer groups). Carers tended to view these as generally supportive and helpful, but felt that a structured, clinician-led approach might be more beneficial. Some carers reported feeling supported or reassured by attending appointments with their child/loved one, though recognized that this was not always possible, if their loved one did not wish for them to attend.

Overall, carers perceived it to be difficult to access support, particularly from clinicians. However, most carers reported finding some value in online resources. Support from friends and family members was additionally recognized as helpful.

#### Negatives of online support

Although carers frequently acknowledged that internet-based support has the potential to be useful (see below), some reported that it may be ‘better for others’ than themselves. Several saw online support as more helpful for those whose loved one had recently been diagnosed, as carers with long-term experience with AN thought that they ‘already knew’ much of the content provided.

Some carers noted that online support might be less relevant for those not caring for their child—it was sometimes perceived to be highly ‘family focussed’, and thus not all resources were as helpful to someone supporting a partner or friend. Additionally, carers mentioned that online support might be more effective and accessible for those who were more frequent users of social media, and enjoyed interacting via technology.

Motivational issues accessing online support were reported by multiple carers, with many stating that (in comparison to face-to-face support), they would not feel as though they were being ‘held accountable’, and would thus be more likely to ‘escape’. Carers felt less likely to ‘open up’ and less able to ‘dig deep’, and reported feeling that it would be harder to develop interpersonal relationships online. Several carers stated that online support could best act as a ‘placeholder’ before accessing face-to-face support.

Carers also reported the possibility of practical issues, including difficulties logging on, potential ‘technical difficulties’, and issues around security and anonymity when chatting with others.

#### Positives of online support

Carers reflected multiple positives associated with online support, including the helpful content, such as dealing with relapse, the thought processes and behaviours of a person with AN, in addition to being reminded of things they had previously learned. Reading the stories and experiences of other carers, being able to ‘pick and choose’ what information to access, and the possibility of self-evaluation/clinician feedback were mentioned as beneficial features.

Carers also listed positives relating to the accessibility/layout of online interventions, including the flexible, low-cost nature, and low ‘entry barrier’ in comparison with accessing NHS support. Related benefits included ease of communicating with others (often perceived to be less ‘intense’ than face-to-face support) and the ability to share content with other family members. Use of multimedia aspects (audio and video), visual aids (e.g. explanatory diagrams), email reminders, and the ability to save progress and return to an online intervention were also described.

Finally, carers reported the positive impact that receiving online support may have on their relationship with their loved one. Some carers reported that their loved one had ‘too much’ going on, and that they might feel ‘guilty’ that their carer required support, so found it helpful that online interventions could be completed privately. Conversely, some carers reported that their loved one may feel happier and more supported, and that accessing an online programme may bring them ‘closer together’.

#### Carer understanding of EDs

A further theme identified by carers was their understanding of EDs—causes, accessing of treatment/support and living with an ED.

Multiple perceived possible causes of anorexia were listed, including societal factors (e.g. ‘skinny as positive’, the beauty/fashion sector, reinforcement via compliments), genetic heritability, and possible links to other disorders, such as depression, anxiety and autism.

In terms of support and treatment, several carers described difficulties for their loved one in accessing NHS treatment and/or acknowledged that their loved one displayed fluctuating motivation for treatment or rejected support. Support from friends was seen as helpful, but with loved ones sometimes being reluctant to seek/accept this.

Finally, carers described some of the difficulties associated with living with AN, including struggles they experienced (e.g. their loved one ‘controlling the kitchen’, getting drawn into arguments), and difficulties faced by their loved one, including the impact of the illness on their relationships, comments on their bodies made by other people, and the detrimental effect on work/studying.

Participants also gave suggestions on the content, features and reach of self-help interventions. The suggestions are summarized in Supplementary table S3 together with the number of mentions among participants.

## Discussion

Study findings give insights into the (i) experiences, (ii) needs and (iii) values and attitudes among people with EDs and their carers regarding ED online interventions; the (iv) facilitators and barriers when optimizing reach, adoption, implementation and maintenance of these interventions; and (v) under-served population groups. While the identified themes did not fit neatly into these groupings, here we discuss our findings in terms of these research questions, so similarities and differences between the two stakeholder groups can be highlighted.

Findings from Study 1 established that most people with EDs were not aware of online self-help programmes for EDs, despite having read self-help books or relevant information online. In Study 2, carers were aware of online resources about AN but thought the information was not detailed or advanced enough to be useful. They also reported not knowing any carer-specific interventions.

Both groups had insight into their need for treatment/support, and saw advantages in accessing online support, including ease of access, overcoming waiting times and receiving support on multiple topics. Study 1 indicated that the accessibility, flexibility and private nature of online self-help may reduce sufferer-specific (e.g. shame, negative attitudes towards treatments) and system-specific (e.g. long waiting times, dismissive attitude of staff) barriers in help-seeking. This is in line with a previous randomised controlled trials (RCT) on online cognitive behavioural therapy (CBT) for people with BN, where 72% of participants had not previously sought statutory help.^14^Participants regarded online self-help as an immediate support and expected to take small steps instead of achieving a full recovery. Taken together, participants’ attitudes and needs support a stepped-care model where online self-help can be a first step. However, consistent with previous literature on people with EDs^13^ and the general population,^22^ participants displayed an interested yet sceptical attitude towards using online self-help. A similar attitude was found among carers—some carers reported that they perceived motivational issues with online support, and that it might at best be a ‘stop gap’. However, overall, carers seemed relatively willing to engage in online support, which may reflect the fact that they are less likely to access face-to-face treatment/support in the NHS. Although offline carer self-help programmes were previously found to be helpful,^23^ engagement was low, possibly suggesting that an online programme may be a more effective way to provide support to carers of individuals with AN.

In terms of increasing intervention reach, participants in Study 1 suggested advertising the programmes directly to users. They noted that groups who are less tech-savvy, such as older people and people with learning disabilities, might find online self-help less accessible. Participants in Study 2 thought online support might be particularly beneficial to carers whose loved one had recently been diagnosed and were a child/family member, and who frequently utilized social media platforms in other contexts.

With regard to facilitators and barriers, in Study 1, two contrasting positions were identified—feeling empowered versus overwhelmed, as well as feeling lonely versus safe, potentially reflecting that many people with EDs experience both facilitating factors as barriers to seeking and accessing support. Overall, participants preferred short, regular engagement with the programme, preferably on mobile apps rather than web-based. Thus whilst web-based self-help usually mirrors face-to-face therapy, e.g. by offering weekly sessions, there may be benefits to different and more flexible approaches to self-help. Carers also had conflicting views around accessing support and frequently reflected their own motivational state as a potential barrier. This is most likely related to the fact that carers typically have multiple demands on their time, in addition to their caring responsibilities. Both stakeholder groups identified similar facilitators for overcoming the barriers, including email reminders, a user-friendly and accessible interface, feedback or monitoring, and engaging with others with similar experiences. Some participants with EDs also identified the importance of having guidance throughout the programme, as well as being given personalized feedback and encouragement. These findings are in line with those from a previous study on the experience of an online self-help programme among people with bulimic-type disorders.^24^

To our knowledge, this is one of the first qualitative studies in the UK that examines both the views from people with EDs and their carers on online ED interventions and support. Some participants were not currently seeking treatment, which facilitated our understanding of barriers of help-seeking. Carers recruited for this study were females and males from across the UK. Qualitative studies allow us to look beyond effectiveness of the intervention and to examine other factors such as facilitators and barriers for adoption and engagement within the RE-AIM framework to better design online interventions. The conversational nature of qualitative interviewing allows us to capture key information that may not have been probed for/enquired into within a standardized survey format. However, the current sample may not wholly represent the views of all people with EDs and their carers, as participants were self-selected and the sample size was small. For example, individuals with AN were not included as self-help is not recommended as a form of treatment due to the higher medical risks.^1^ Additionally, as the researchers engaging in qualitative interviewing were directly involved in the running of online self-help interventions, this may have evoked the presence of demand characteristics during the interviewing, and perhaps social desirability bias, which may have prompted participants to speak more favourably about the merits of online interventions. Future studies could include other stakeholders such as healthcare providers to explore their views on implementing online interventions for this population in routine healthcare settings. Other methodologies such as a questionnaire survey and usability testing could be employed to increase sample size and to examine usability factors such as user experience in-depth to complement the current findings.

The findings reveal the different expectations and functions of online self-help perceived by people with an ED and their carers. Both groups are likely to be open to using online interventions to support their recovery/enable them to support their loved ones effectively. Nevertheless, identified barriers require further exploration and it may be helpful to consider a ‘stepped care’ approach for people with ED and their carers.

This study lays a foundation for future design and evaluation of online self-help programmes, in terms of the values, attitudes, facilitators and barriers perceived by different stakeholders.

## Supplementary data

Supplementary data are available at *EURPUB* online.

## Funding

This project has received funding from the European Union’s Horizon 2020 research and innovation programme under grant agreement no 634757. L.S. is supported by a PhD studentship, funded by the European Commission—Horizon 2020 programme. U.S. is supported by a Senior Investigator award from the National Institute for Health Research (NIHR) and receives salary support from the NIHR Biomedical Research Centre for Mental Health, South London and Maudsley National Health Service (NHS) Foundation Trust and Institute of Psychiatry, Psychology and Neuroscience, King’s College London. The views expressed herein are not those of NIHR or the NHS.

*Conflicts of interest:* None declared.

**Key points**

- People with BN and BED, and carers of people with AN report having some awareness of existing online resources, but few had engaged with this form of support.
- Both groups were able to identify both positive and negative aspects of utilizing online support, with the majority indicating that they believed it could be a useful aspect of their respective care pathway.
- These findings are in line with the current NICE guidelines suggesting that individuals with BED and BN may benefit from self-help. Online support could be a potential service provision due to its scalability and accessibility to a broader population.
- Carers are a frequently under-served population, and there are few recommendations given with regards to how they may benefit from support. It may be helpful for future NICE guidelines and healthcare policies to consider recommending online support for carers, as this may be a useful and acceptable way to support their own mental wellbeing, and indirectly, that of their loved one.

## Supplementary Material

ckab046_Supplementary_Materials
