# Supplementary material for: Views on online self-help programmes from people with eating disorders and their carers in UK
Source: Eur J Public Health. 2021 Jul 7;31(Suppl 1):i88–93. doi: 10.1093/eurpub/ckab046 (PMC8495677; doi:10.1093/eurpub/ckab046)
Supplement: ckab046_Supplementary_Materials [file ckab046_supplementary_materials.pdf]

Supplementary Materials

Views on online self-help programmes from people with eating disorders and their carers in UK

Yim SH, Spencer L, Gordon G, Allen K, Musiat P, Schmidt U.

Appendix 1 Topic Guides

<u>Study 1: everyBody Plus Stakeholder Survey Focus Group/ Telephone interview</u>

Focus Group Topic Guide (Target Group- individuals with a diagnosis of Bulimia Nervosa and Binge Eating Disorder.)

KEY TOPICS

Warm up (refers to "needs") [Research Question 3]

1. What do you think are the biggest mental health challenges for the health system in your country?
    a. … for patients with eating disorders?
    b. … for patients with eating disorders on a waiting list for psychotherapy?

[Relevance of topic "self-help to bridge waiting time for treatment" in general]

[Question 2 is assigned to Research Question 4 (Values and Attitudes)]

2. How do you feel about self-help interventions (to bridge waiting time for the treatment of eating disorders) in general?

g. Do you have experience with self-help intervention?

[Experiences with internet-based interventions]

[Research Question 2]

3. What are your experiences with Internet-based self-help interventions?
    - Which Internet-based self-help interventions are available in the health care system in your country? [name, age group, focus]

[Values and attitudes]

[Research Question 4]

4. What do you think about Internet-based self-help interventions for patients with eating disorders in the health care system in your country?
    a. What do you consider as the most important advantages of Internet-based self-help interventions compared to face-to-face interventions (like self-help groups)?
    b. What do you consider as the most important disadvantages of Internet-based self-help interventions compared to face-to-face interventions (like self-help groups)?

[Needs]

[Research Question 3]

1. Which topics need to be focused on when offering Internet-based self-help (for patients with eating disorders) in the health care system in your country?
    a. Which overall aims need to be focused on?
    b. Which characteristics would you consider important for Internet-based self-help interventions in the health care system in your country?
    c. Which groups of individuals would you consider most underserved regarding Internet-based self-help interventions?

[Barriers and facilitators for reach, adoption, implementation, and maintenance] *

[Questions 3 and 4 are assigned to Research Question 5]

[REACH]

2. Which factors need to be considered to make Internet-based self-help interventions (for patients with eating disorders) attractive?

[IMPLEMENTATION]

3. Which factors need to be considered that Internet-based self-help can be integrated in the daily routine of individuals?

[CONCLUSION/ END]

4. Is there anything else you would like to tell us?

<u>Study 2: We Can Stakeholder Survey Focus Group/ Telephone interview</u>

Focus Group Topic Guide (Target Group - individuals caring for a person aged 14+ with Anorexia Nervosa)

Focus Group Topic Guide (Target Groups)

KEY TOPICS [Add probes for fuller response and/or change order depending on situation and participants]

Warm up (refers to "needs")

1. What do you think are the biggest mental health challenges for the health system in your country for people with an eating disorder?
2. How do you feel about self-help interventions in the field of mental health in general?

[Experiences with Internet-based interventions]

3. What are your experiences with Internet-based interventions that aim to prevent mental health problems and disorders?
    a. Specifically as a carer?
4. Which Internet-based interventions that aim to prevent mental health problems and disorders are available in the health care system in your country?
    a. Specifically as a carer?

[Values and attitudes]

4. What do you think about offering Internet-based interventions that aim to prevent mental health problems and disorders within the health care system in your country?

a) What do you consider as the most important ***advantages*** of Internet-based interventions that aim to prevent mental health problems and disorders compared to face-to-face interventions?

- Any carer-specific advantages?

b) What do you consider as the most important ***disadvantages*** of Internet-based interventions that aim to prevent mental health problems and disorders compared to face-to-face interventions?

- Any carer-specific disadvantages?

[Needs]

5. Which topics need to be focused on when offering Internet-based interventions for carers?

a) Which overall aims need to be focused on?

b) Which characteristics would you consider important for Internet-based interventions for carers?

c) Which groups of individuals would you consider most underserved regarding Internet-based interventions for carers?

[REACH]

1. Which factors need to be considered to make Internet-based interventions attractive?
    a. Specifically for carers?

[IMPLEMENTATION]

2. Which factors need to be considered in order to successfully integrate Internet-based interventions in the daily routine?

[CONCLUSION/ END]

8. Is there anything else you would like to tell us?

Appendix 2. Illustrative quotes

Table S1: Illustrative Quotes for Theme and Subthemes in Study 1

Theme 1. Barriers affecting help-seeking behaviour	
Sub-themes	Illustrative quotes (Participant Number)
Prior experience	"My perception was that because the NHS has long waiting time there's really no point." (P11)<br><br>"It felt dismissive I guess, erm, and then years and years later when I first went to the therapy… that was a 4-5 months CBT thing, well the person said to me was to be expected someone much fatter…this is in a way that I found it so upset. I felt so sad that my eating was so out of control and I can't stop, and she was like, you don't like you have a problem, and again dismissive" (P9)
Self-stigmatisation	"When I was younger, I didn't realise it was the actual problem, kind of, ignoring it or something, at the same time, this is kind of life the, kind of just a fat girl…I just need willpower to deal with it." (P12)
Internal attitudes	"They know about bulimia and that aspect and that kind of things. But if you don't fall into that category, then you are just a pig, and weak or whatever it is." (P12)

	"I think that would be, especially for the binge eating, and bulimia, it's so compared to anorexia, you are just…I have always been made to feel like it's not as big an issue, it's not as immediate as anorexia. I feel that that's the glamourous one, and that's how the media portrays it, it's just just so embarrassing, not being good enough of even having the eating disorder." (P3)<br><br>"I think that from my experience, with eating disorders, there is a part of the eating disorders that doesn't want to let go, and I think that can be a barrier because for people to really give up their eating disorder, this is a very difficult, very lengthy, long. It requires a lot of sacrifices." (P5)
Theme 2. Attitudes towards self-help	
An option but not a replacement	"I would definitely use it, especially if my motivation would (be) high enough to you know, actually improve, which I guess the time of seeking treatment is like, the highest, but I am not sure how sure how much faith I have in self-help? Given that you know the problem, kind of starts within the self….the fact that I am seeking for help kind of

	means that I, on my own, can't really do enough when I was sick.” (P7)<br><br>“It might be an advantage that it gives someone the opportunity to reflect themselves on some of their problems, maybe before entering therapy, they get a clearer idea of what their problems are and potentially getting some short-term ways of dealing with them.” (P14)
Works differently for different people	“If I haven’t grown up from this like people of my age or younger…they can find it difficult to understand how to use everything, erm, how to navigate things, how to communicate what is the difference between the forum and the chat room and the private message might be that kind of thing” (P9)<br><br>“I guess adolescents have less of a, a bit more difficult for adolescents to stick to it and use it, and have the self-determination or motivation to actually stick to a self-help programme. I think they could be more helpful for adolescents, eh, for adults, and less helpful for adolescents or children who might need a bit more supervision.” (P7)

Empowerment and overwhelming feeling	"It's a benefit of having it available and being able to decide for yourself of what and when you are need to use rather than like, the group therapy, it's like each week there's a set topic, so some weeks there are more relevant than others… whereas when it's all online, you get to pick and choose." (P13)<br><br>"How you can do it at any time and process it slowly and I think there's so much information that you take in…I guess I will benefit from online resources that like, you can absorb more of the information and go back to it, and that's like, the good things." (P1)<br><br>"You are having to confront more painful things, and you have to be more honest for yourself, and the tool might do that even more, (e)specially if somebody doesn't have a support network, or somebody to talk it through with, this can be a bit isolating." (P14)

Loneliness and safety	"Yeah, because machines really don't give a shit if you don't do it, what are they gonna do they don't mind." (P12)<br><br>"you have a diversity of people that you speak to, have some men, have some young people, old people, trans people, that they are included…<br>it's the eating disorders specifically so I am often sceptical about because all of the imagery and visual about it are of very thin white girl and I am an obese black woman, I do not feel represented, I do not feel these things are helpful for me, because I do not feel represented in the imagery…" (P9)<br><br>"there is a community aspect, but it needs to be moderated…it's far too easy for the wrong kind of people to get into these sorts of groups. I tried to go on a binge eating help group on Facebook, saw people who were very, very slim saying, they binged on a whole yogurt...there was clearly something going on in their head, but it's not binge eating, whatever they were going through…make sure that it's a safe and welcoming environment for anyone who wants to use it would be important." (P13)

Theme 3. Engaging with the online self-help intervention	
Baby steps	“it's like a slow progress, and then it’s like baby steps rather than, there aren’t any instant things that you can get better, but sort of like know they will take a bit of time, more like the little smart goals, maybe at the beginning it will be good to do like, set yourself like these small goals, as opposed to like the huge goal of like getting rid of the eating disorder.” (P2)<br><br>“How to pace things, this is something I've learnt elsewhere that I've applied to my therapy that has been helpful. While I wasn't feeling like you have to jump in to do the three meals and snacks a day thing, how to just say like, okay, so I am gonna start out by just making sure that I have breakfast every day…like building it up, slowly, so that you are not just trying to overhaul your entire life in a week.” (P13)
Immediate support	“If I can press a button and get immediate help, you know, sort of 30 seconds thing, and then, press it again, maybe I will get another one, or

	maybe a longer meditation, or maybe if I also a bit more, and it takes me to a story that would take 2 minutes to read. You know, something like that, I choose how much, it could be how distressed you are on a scale of 1 to 10, you know, I am an 8, or how strong is the craving, it's 5…that would automatically take you to sort of something that was gonna help in that moment." (P12)<br><br>"The benefit of online support is always there, like sort of one o'clock in the morning, and I can't sleep, and I'm having intrusive thoughts, then I've got things there to support me, where face to face therapy and group therapy happen once a week at a regular time, or doctor's appointment, you may not be able to get one where you need one" (P13)
Monitoring and feedback	"it has to be something in the app, you would love so much, it would reward you for logging in to log your food plan or whatever you will need to do, would be so rewarding that I can kind of think at the moment, well done well done, that, even if you haven't done your food plan or something, you don't have to face the fact that you haven't done it" (P12)

Easy to use and be integrated to daily life	“Definitely if it's app-based…like the [recovery record app] that reminds me to eat regularly. That's so, so helpful, I can't stress enough that would be a big factor in getting it integrated into people's lives.” (P13)<br><br>“Perhaps something like a time where you want, where counsellor will actually contact them to update their progress, or you know, if someone hasn't updated them for long, then the system or something would ask them, "oh, you know, you haven't been updating for long", but then again, not that kind of generic you know reminder messages, "oh you haven't been logging in for how many days" that kind of message, that you actually feel like someone worries about you, and things like that.” (P6)

Table S2: Illustrative Quotes for Themes and Subthemes in Study 2.

**Theme 1. Current status of existing ED carer support**	
Subtheme	*Illustrative quotes*
Online support	"I know there are things like Beat and Mind websites, and I do follow some on social media. Some accounts on Instagram and things where they post." (P6)<br><br>"Mostly they seem to have like quite basic information, which maybe, maybe that's more helpful if you're kind of just starting out. It would be better for there to be some more like, detailed stuff. I do try and read news articles and things, but it would be better to have on more websites I think." (P11)
In-person support	"I've been part of a kind of support group, that I went to on two or three occasions at the initial clinic that X was supported… which was basically… where parents could get together and talk. To be honest it wasn't very helpful because, um, I found, if I'm honest, that I was almost - I found myself leading the group." (P2)<br><br>"You kind of get left a bit because the clinics and things obviously, sadly aren't as accessible around… [the Christmas period]" (P5)
**Theme 2. Negatives of online support**	

Better for ‘other people’	“I do think it would be more helpful to someone who might be more, less further along in their ‘journey’… I think for me it isn’t so much, because lots of the information are like things I already know, or figured out.” (P11)<br><br>“Online things I think can work yeah, but maybe some people find them more relevant. They seem quite, to be quite family focussed a lot of the time, which isn’t really as relevant to me. I do appreciate it but I think seeing a real person, I think I would prefer seeing a real person.” (P1)
Lack of motivation	“I think seeing a professional face to face is going to be better than doing something online. I suppose it's like having a conversation with anyone face-to-face is always a better thing to do, rather than sending an email to somebody. You get more of an actual type of relationship.” (P10)<br><br>“You're usually kind of pushed to attend as well, because there is someone there, someone telling you whether you have missed a session, or... there's someone kind of keeping a bit of a tab on you. Whereas with the self-help things you don't necessarily have to do them. So you can kind of get

	away with not doing it, and that's not going to help you in the long run." (P7)
Practical issues	"Well you can have all the like, technical issues I know. If the internet's not working when you need to be on… that can be an issue for online, or if it's hard to use website, or to type in." (P3)<br><br>"Maybe you'd be worried about being anonymous when you're talking. Like if it's secure or if there might be some issue with people knowing who you or your child is." (P12)
**Theme 3. Positives of online support**	
Content	"I liked the fact that, you know, as well as kind of, making the point to use characters and scenarios to, to show those points, most of the scenarios I could relate to very easily." (P2)<br><br>"It's good because you feel that you're not alone, you know, and obviously the different topics and the forums are quite useful because you can find the one that relates to you at that particular time if you like, and I did find that really helpful." (P9)<br><br>"You can get reminded of things you do already know, but you might, you might forget. Like a reminder, a

	reinforcing of things you know. Then you can look back too at times when it's worse… maybe to check and get feedback of where you are now." (P4)
Accessibility and layout	"I suppose with the internet, internet self-help it's accessible to everybody, not everyone may be able to afford to go to a counsellor, or they might not, within the NHS they might not classify to receive counselling under the NHS for whatever reason, so." (P1)<br><br>"It's good that you can get the reminders, so then you can just logon and get started. Then you can have like um, the different aspects so, the videos or things you can hear… and where you can leave messages for the other people." (P6)
Impact on relationship with loved one	"I think overall she would, I think if she was of sound mind, and thinking sort of, um, kind of rationally, she would, she thinks it's positive, a positive sort of experience. She's not been negative in any way, but I kind of would be very careful about when I have a discussion about something that I might've done." (P5)<br><br>"I do think it brought me closer to her, she was glad. It's like now we can talk more about things, even that

	some of, some of her other friends who are closer can't talk about to her." (P8)<br><br>"I guess for some people they might not want to tell them, put too much stress and feeling guilty on them. But no, yeah I think for me it's positive, even though she didn't really get much out of it herself." (P1)
**Theme 4. Carer understanding of EDs**	
Causes	"Reinforcement in society that… I suppose it's in lots of things, like it's in the kind of like beauty sector, I suppose." (P12)<br><br>"Images in society, all a bit sort of skinny, healthy eating, all those sort of pressures that are in society anyway, and for people that have got anorexia would be even more in their face, the fact that being skinny is a great thing." (P6)<br><br>"In my friends situation, men are then like, that is how a woman should be, then when they come into contact with somebody they might not know that they have anorexia, and they will then reinforce that that they look skinny, and they will only, I suppose, make things worse. They may voice those opinions too, like 'Oh you look really beautiful' but actually,

	they don't realise that that is actually quite a big thing for the person to hear, and it is not the best thing to hear if they've got that sort of illness." (P8)<br><br>"Also with like, it can be part of your genetics, and can maybe be linked with other things I guess, with being depressed. Or apparently it might be to do with autism in some way, I think I read." (P2)
Accessing support and treatment	"It can be really a, quite a long and hard process, to actually get to, to the point of really getting some support." (P10)<br><br>"Sometimes they really don't want help, especially when it's inpatient. On different days X would sometimes be like yeah, really positive… some days she would just not at all want any help. It really depends." (P7)
Living with an ED	"Getting drawn into arguments… if they're sort of, in charge of the kitchen it's hard to have a conversation about that without it being an argument… the 'anorexic voice' is there and then it's so easy to become an argument." (P2)<br><br>"I think hearing other peoples' comments, it has such a big impact. It's already pretty, so hard to live a

	normal life for them, with University or maybe working, and other people say things… it makes X feel even worse that other people are saying things and, talking about her." (P8)

Table S3: Suggestions given by Participants on Online Self-Help Intervention Programmes

	Illustrative quotes	Frequency of mentions
**Study 1: People with an ED**		
Cognitive-behavioural technique		
1.1 Emotional management such as anxiety management and distress tolerance	"When people don't stick to their plan, they have to be a kind of, okay, what happened before, right, okay, so you are upset by this, asking lots of questions, again if you can do that from an app. yeah, I guess, you can just report on the app and then the app maybe able to tell you back and suggest, you know, try to set aside what helps you had to deal with whatever the emotion at that point that kind of thing." (P12)	4
1.2 Distraction technique	"So like, set yourself little challenges, name 5 things that begin with the letter S, or you know, think of given that you sort of the alphabet, or find somebody and give them a genuine compliment, or find 3 things around you that you would like to do by yourself. I don't know, these would be really good for an app, you know, I am feeling a craving, I want to go and buy chocolate or whatever it is, and it could immediately challenge for you, and you will get distracted in that moment. That might be quite useful. And you know craving is kind of hard, and they are gone, and the emotions, you can distract yourself" (P12)	4
1.3 Mindfulness	"Bring out the concept of mindful eating …instead of not eating and not really interrogating why to think I am actually hungry if I	4

	am hungry then I should eat… why am I doing this what am I feeling if I am feeling anything … or is it automatic erm yeah mindful eating I think, it is really important concept that can do to people" (P9)	
1.4 Thought challenging	"If there's an inbuilt tool that you can just go to quickly online that would help you for example work through a negative thought process, maybe there is something in the system that helped you identify the thoughts, and hm, you know, come up with alternative thoughts or something. I can imagine there would be some practical tools that you could do on your own and there's some structure there already, for you to think about it." (P14)	2
Eating disorder-specific advice		
2.1 Signposting and directory	"Also, maybe other platforms where to find easily contacts with one more face to face approach, to gather all the parts about information, about yeah. Just a list of emails of people, centres you can refer to, possibly it would be important." (P10)	7
2.2 Dietary advice	"It's a bit hard because I guess if you are doing it for really long, you don't really know what a portion size is like anymore, so you just eat whatever that's from your sight while trying to learn more about how much you actually eat, I guess, things like that, on how to slowly revert that to a normal eating cycle. That would be helpful." (P6)	6

2.3 Practical rather than generic advice	“I guess it's just to tell people what they already know. Hm, I don't know, like, kind of that kind of general article, or self-help book, like "oh, you should go for a walk", I don't know, that kind of generic articles where they ask you to relax, or things like that, things that don't really help.” (P6)	6
2.4 Causes underlying ED	“I think the focus should not be too much about the food because I think it's not about the food. Eating disorders specifically food, it will be simple to deal with them, but instead, it's not about the food, it's about all the underlying issues, and that's really hard and they are really naughty, and I think you need to make something that deal with thoughts that come up, or, things that try and you know get people to understand why they are doing what they are doing with food.” (P5)	5
2.5 Psychoeducation about ED	“maybe even like some literature of something to accompany the tool, if there's something I can read beforehand, for me to get to know why this is important, and what exactly is being tracked” (P14)	3
2.6 Psychoeducation for family and friends	“for other sections which my friends can go there and have a read. Maybe for me it's difficult to talk about my problems. So rather than me explaining all the parts, they can go and read a bit about it.” (P10)	2

2.7 Offline materials	"Printable material, because I've been copying and pasting stuff to keep it for the future" (P2)	2
3. Reliable source	"I think if it's evidence-based, like, have more time for it, cause there are lots of rubbish on the internet, you know, and I think having something that is like, stop wasting your time on all these other stuff, and get one... You know, I have watched Youtube of a lot of people with their cats talking, you know what I mean, like, I think you've got to separate them, and I think having something that is medically, this is shown, this is researched, this works" (P5)	6
4. Sensitive branding	"There's a lot of shame around that, it takes a lot for someone who's struggling with binge eating, and I think it's really important that that is addressed, so I think the way it is advertised needs to be like, look it's okay for you to have this problem, so does so many other people, because they do, but I think when you are in it, it can feel like you are the only one, but you are not, and you are not alone, so I think that could be really helpful to know" (P4)	3
5. Advertising beyond the healthcare system	"If it's one of the first things that came up on Google, that would, you know, if it was just there right away, as an option, and you didn't have to search very hard for it, you won't have to go there, have to think about those things, you can deal with those issues." (P15)	2

	"Before maybe even going for the doctors, there was also a way that you could have a therapist, like online questionnaire-type thing, or phone call or some kind of way of diagnosing someone and giving them a course before even going to the GP." (P4)	
**Study 2: Carers**		
Content		
1.1 Stories and case studies	"Everybodys' journeys are individual and, who knows how things pan out, turn out. But, I think maybe if there were some case studies, or family experiences that relate to how long that could be, again it might give people the opportunity to think more realistically about living with this." (P4)	7
1.2 Detail	"I do think, being more detailed, definitely. 'Cause I know there are things out there, websites and things, but they often just say oh, this quite basic thing that everybody knows if they've been caring for somebody. The information is good and it's not wrong, but I do think more detail would be helpful, especially if you've been dealing with it for a while." (P11)	6
1.3 Interactivity	"Maybe they could be a bit more interactive, I don't know. there could be examples that pop-up like as the diagram kind of brings up... and then I suppose like a voice saying what an example of that could be." (P3)	6

	"I suppose I'm much more of a visual person than text. I think that videos, where you can just see and listen, kind of with characters and scenarios to show you conversations." (P2)	
1.4 Starting conversations about change	"Sort of plans of how you're going to talk to your child or whoever. I suppose trying to generate a conversation, like help us to start conversations about how she was feeling. Because I think sometimes it's quite difficult to know how to ask somebody how they are, you kind of don't want to just come out with something. I think maybe something about positioning questions in a certain way to have the, the hard conversations about her changing." (P10)	4
1.5 Further resources	"So maybe gathering, and sharing you know, sort of, useful books, documents… yeah yeah, sort of like a resource bank of, of kind of recommended resources maybe?" (P8)<br>"Being able to have something printed, I mean for me that would be helpful... stick it on the fridge or something, so you can see it when it's helpful, and when you don't, you're not necessarily always at your computer or your phone." (P2)	4
1.6 Exercises to complete with loved one	"Maybe some exercises for the carer to go through with their friend, or whoever, like from her counsellor she has been given a workbook and we were working through it the other day. I do think yeah, something like that would be helpful, and you'd be working together and maybe get closer." (P8)	3
1.7 Feedback and monitoring	"I do think that some feedback... where you are now, how you've improved from before, maybe telling you back your scores on	3

	things. I guess you could have things that you fill and it like, feeds that back to you, or you could have someone kind of on the other end." (P7)	
Accessibility		
2.1 Usability	"I guess it's got to be easy to use, and a bit, appealing to look at... not too much text all in a row, break it up with pictures and videos or something." (P4)<br>"I do think being able to have a break, and like save the place, save where you are, and then come back to it later would be good." (P9)<br>"Being able to chat to people I would find really helpful, but as long as it works, maybe like a Facebook style thing, to click on and be able to chat to people." (P5)	8
2.2 Mobile phone / app-based	"Well I think, having the app is a good idea. People are not always at a laptop or desktop or whatever." (P12)<br>"Probably, once you've got an app running, it probably will be easier for people to manoeuvre...<br>and probably, because of the nature of apps, people kind of see and click on it a bit easier." (P6)	5
2.3 Publicising of intervention	"I would definitely say it should be available for everyone who wants to, like, through the NHS, and advertised through them so like, when your daughter is getting treatment they could also be like 'there's this programme for carers if you want to', and you can log on and do it while they get treatment." (P5)	4

2.4 Podcasts and ‘seminars’	“I suppose you could have like podcast type things, you could maybe like listen to it, rather than on a computer or whatever. You could just listen to it rather than actually having it open on the phone.” (P12)<br>“In my work I sometimes do online seminars. that could be something, people could listen to them.” (P2)	2
